# Supplementary material for: WNK1 Kinase Stimulates Angiogenesis to Promote Tumor Growth and Metastasis
Source: Cancers (Basel). 2020 Mar 2;12(3):575. doi: 10.3390/cancers12030575 (PMC7139507; doi:10.3390/cancers12030575)
Supplement: Supplementary file 1 [file cancers-12-00575-s001.pdf]

Supplementary Materials: The following are available online at www.mdpi.com/xxx/s1,

Materials and Methods

To determine the optimal concentration of WNK pathway inhibitors for xenotransplantation assay, 3 dpf zebrafish embryos were immersed with serial dilution of WNK463 or Closantel for two days. Embryos exhibited 100% survival rate at 7.5 μM WNK463 and death at progressive higher concentrations of WNK463 (Figure S3A). The LC_{50} of WNK463 was calculated at 11.21 ± 0.46 μM (Figure S3A). Abnormality of embryos yet were noticed at 7.5 μM WNK463 (Figure S3B). The final concentration of WNK463 used for xenotransplantation assay, thus, was reduced to 2.5 μM. Compared to WNK463, Closantel was more toxic (Figure S3D). The LC_{50} of Closantel was calculated at 0.1913 ± 0.000 μM (Figure S3E). The safety margin for Closantel is narrow: survival rate of embryos were only 17% at 0.2 μM (Figure S3F). Thus, we decided on 0.15 μM as optimal concentration of Closantel for the xenotransplantation assay.

The therapeutic index (TI = LC_{50} / IC_{50} [1]) was calculated to be 0.21 and 0.09, respectively, for WNK463 and Closantel, suggesting that WNK463 is a safer therapeutic drug than Closantel assuming translatability of the fish results to humans.

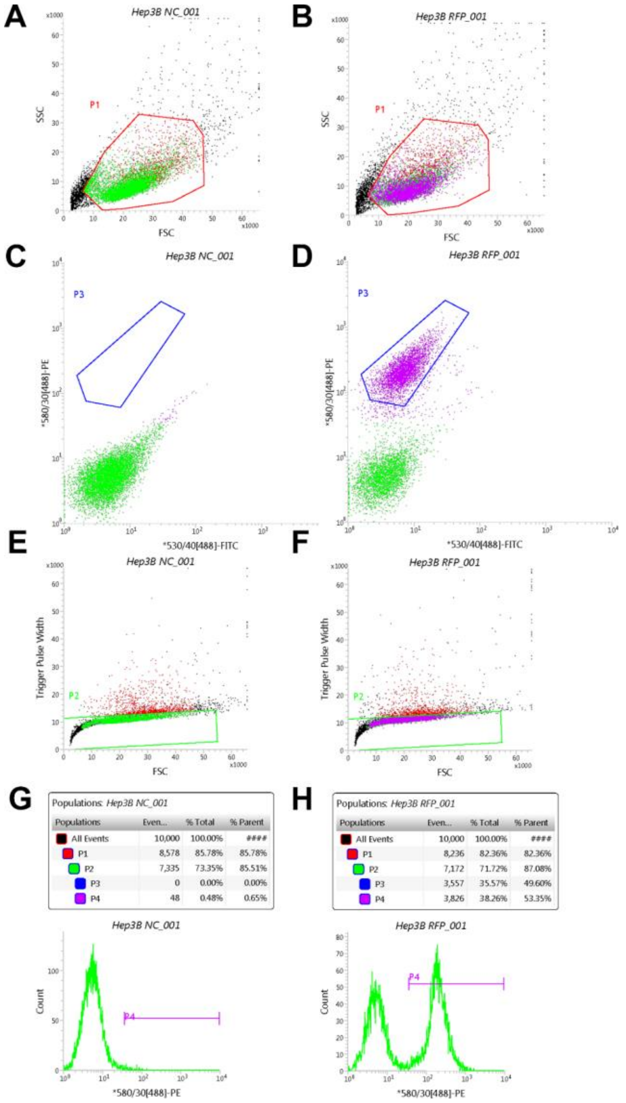

Figure S1. FASC of un-transfected and rLVUbi-LifeAct-TagRFP transfected Hep3B cell lines. (**A, B**) Forward and side scatter plot of un-transfected (A) and transfected (B) Hep3B cells. The x-axis represents forward scatter (FSC) where an increased signal can indicate increased cell size. The y-axis represents side scatter (SSC), where an increased signal can indicate increased cell granularity. Un-transfected and transfected Hep3B cells were in the same scatter gate (red line), shown that Hep3B cells did not change cell size or granularity after transfection. (**C, D**) Singlets discrimination (FSC versus trigger pulse width) plot of un-transfected (C) and transfected (D) Hep3B cells. (**E, F**) Comparative dot plots plot of un-transfected (E) and transfected (F) Hep3B cells. The x-axis represent yellow-green fluorescence signal into the FITC (fluorescein isothiocyanate) detector. The y-axis represent red fluorescence signal into the PE (fluorescein) detector. The green and purple population indicated fluorescence differences between wild-type and RPF+ cells. The purple cell pools (blue line) were sorted to create stable Hep3B_LifeactRFP cell line. (**G, H**) Histogram and sorting gates of un-transfected (G) and transfected (H) Hep3B cell.

Formatted: Font: Bold

Formatted: Font: Bold

Formatted: Font: Bold

Formatted: Font: Bold

Formatted: Font: Bold

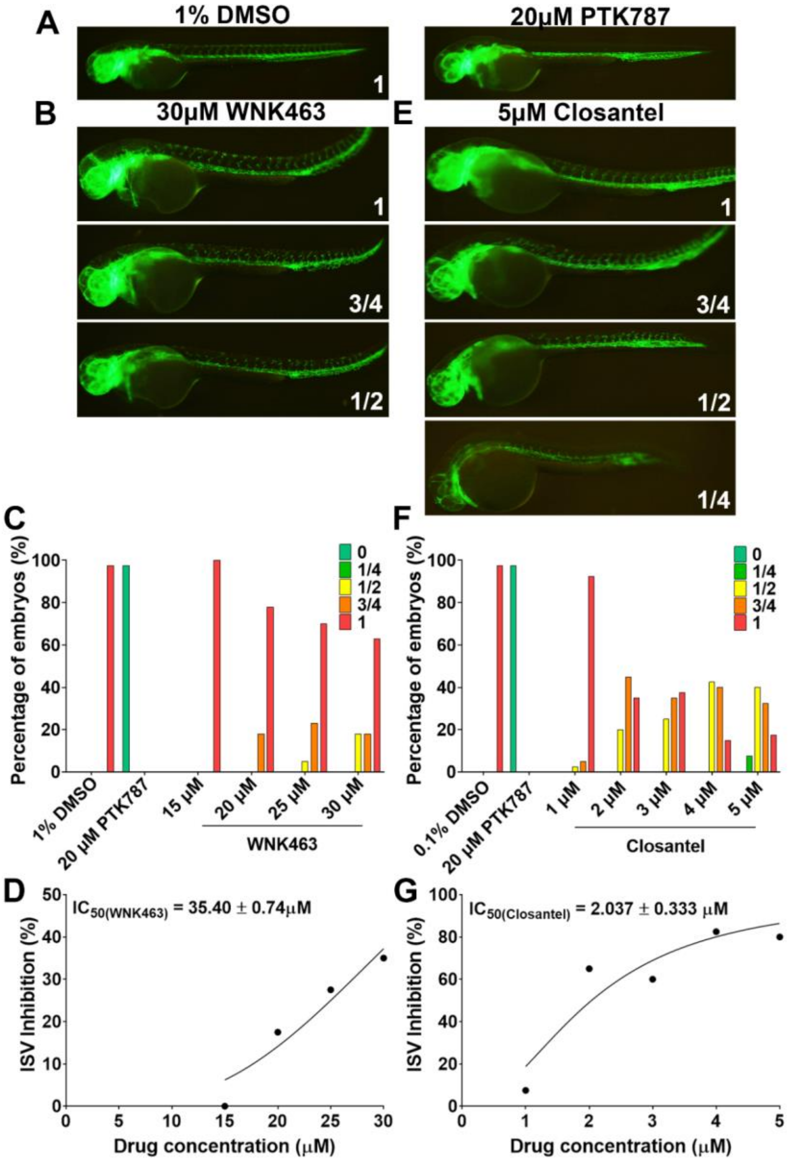

Figure S2. Anti-angiogenic effect of WNK463 and Closantel. (**A**) Representative images of *Tg (fli1:EGFP)* embryos immersed with vehicle (DMSO), VEGF inhibitor PTK787 or WNK463. Embryos were immersed with drugs at 1 dpf, and observed for the effects on the length of the intersegmental vein (ISV) for subsequent 24 hours. ISV length of the embryos treated with WNK463 was measured and classified in 5 groups: full length (1), three-quarter length (3/4), half length (1/2), quarter length (1/4) and no-ISV growth (0). (**B**) Distribution of ISV length from embryos treated with different concentrations of WNK463. (**C**) Dose-response inhibition curve of WNK463. (**D**) IC50 of WNK463. (**E**) Representative images of *Tg (fli1:EGFP)* embryos immersed with DMSO, VEGF inhibitor PTK787 or Closantel. (**F**) Distribution of ISV length from embryos treated with different concentrations of Closantel. (**G**) Dose-response inhibition curve of Closantel. (J) IC50 of Closantel.

Formatted: Centered

Formatted: Font: Bold

Formatted: Font: Italic

Formatted: Font: Bold

Formatted: Font: Bold

Formatted: Font: Bold

Formatted: Font: Bold

Formatted: Font: Bold

Formatted: Font: Bold

Formatted: Font: Bold

Formatted: Font: Bold

Formatted: Font: Italic

Formatted: Font: Bold

Formatted: Font: Bold

Formatted: Font: Bold

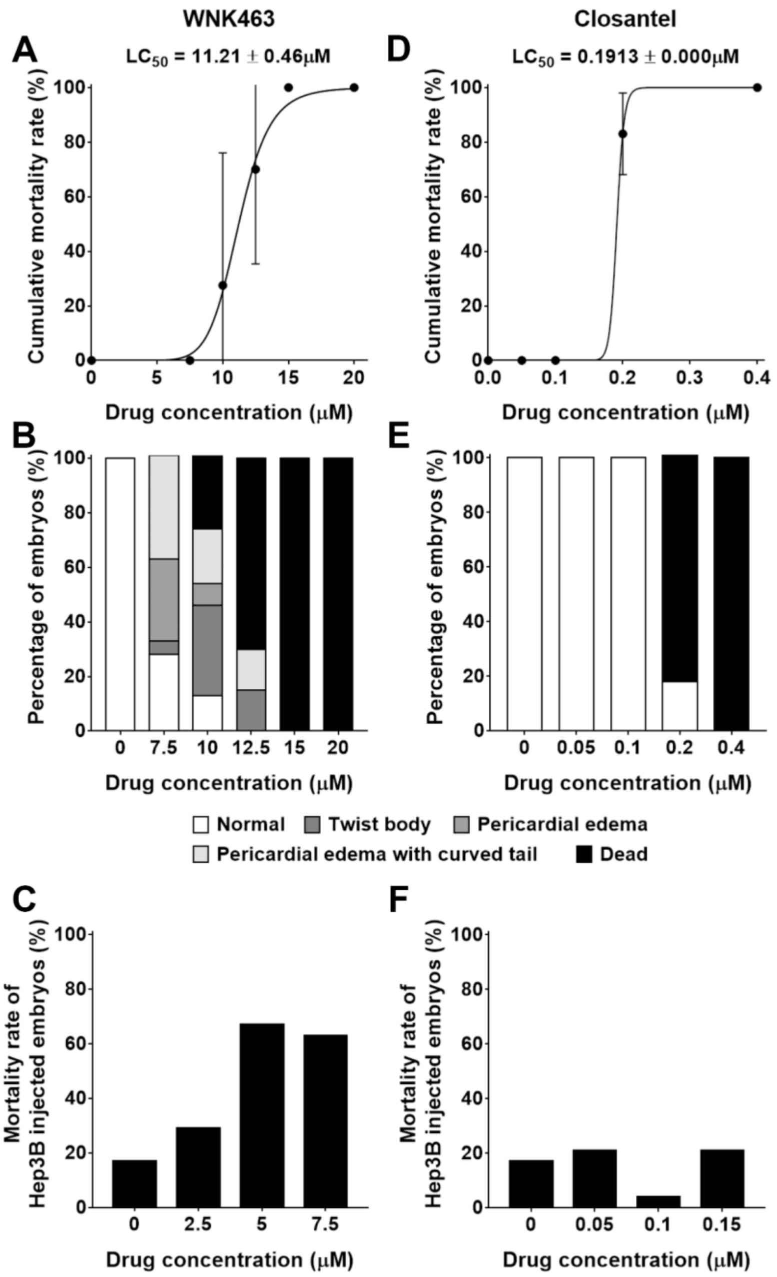

Figure S3. Survival test and determination the LC50 of WNK463 and Closantel for xenotransplantation assay. (**A**) Cumulative mortality rate of 5 dpf embryos treated with WNK463, and LC50 of WNK463. (**B**) Distribution of phenotypes of 5dpf embryos treated with WNK463. (**C**) Mortality rate of Hep3B injected embryos treated with WNK463. (**D**) Cumulative mortality rate of 5 dpf embryos treated with Closantel, and LC50 of Closantel. (**E**) Distribution of phenotypes of 5dpf embryos treated with Closantel. (**F**) Mortality rate of Hep3B injected embryos treated with Closantel.

Formatted: Font: Bold
Formatted: Font: Bold
Formatted: Font: Bold
Formatted: Font: Bold
Formatted: Font: Bold
Formatted: Font: Bold
Formatted: Font: Bold
Formatted: Font: Bold

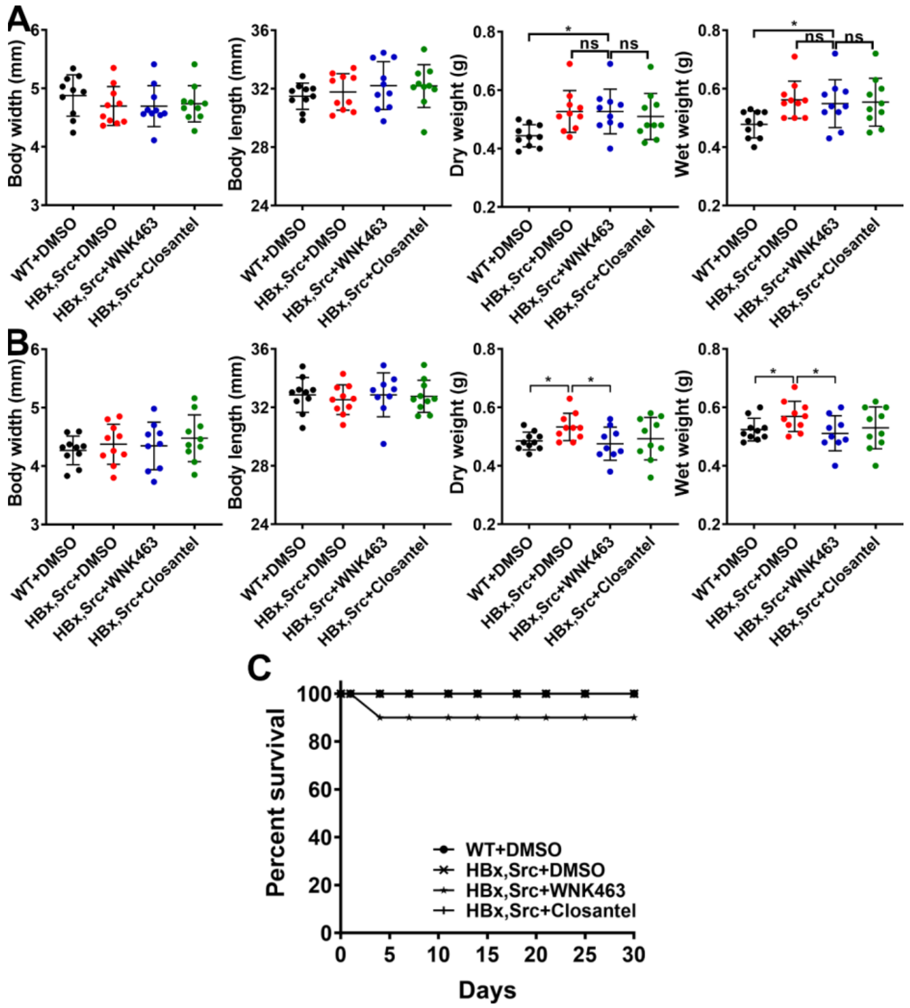

Figure S4. Body width, length, weight, and survival of transgenic hepatoma fish before and after oral gavage of WNK pathway inhibitors. (**A, B**) Body width, length, and weight of 5-month-old *Tg (fabp10a: HBx-mCherry, src; myl7:EGFP)* fish before (A) and after (B) treatment with DMSO, WNK463 or Closantel. *: 0.01<P≤0.05; **: 0.001<P≤0.01; ***: P≤0.001. (**C**) Survival curve for WT fish and Tg hepatoma fish with drug treatment.

Formatted: Font: Bold

Formatted: Font: Bold

Formatted: Font: Italic

Formatted: Font: Bold

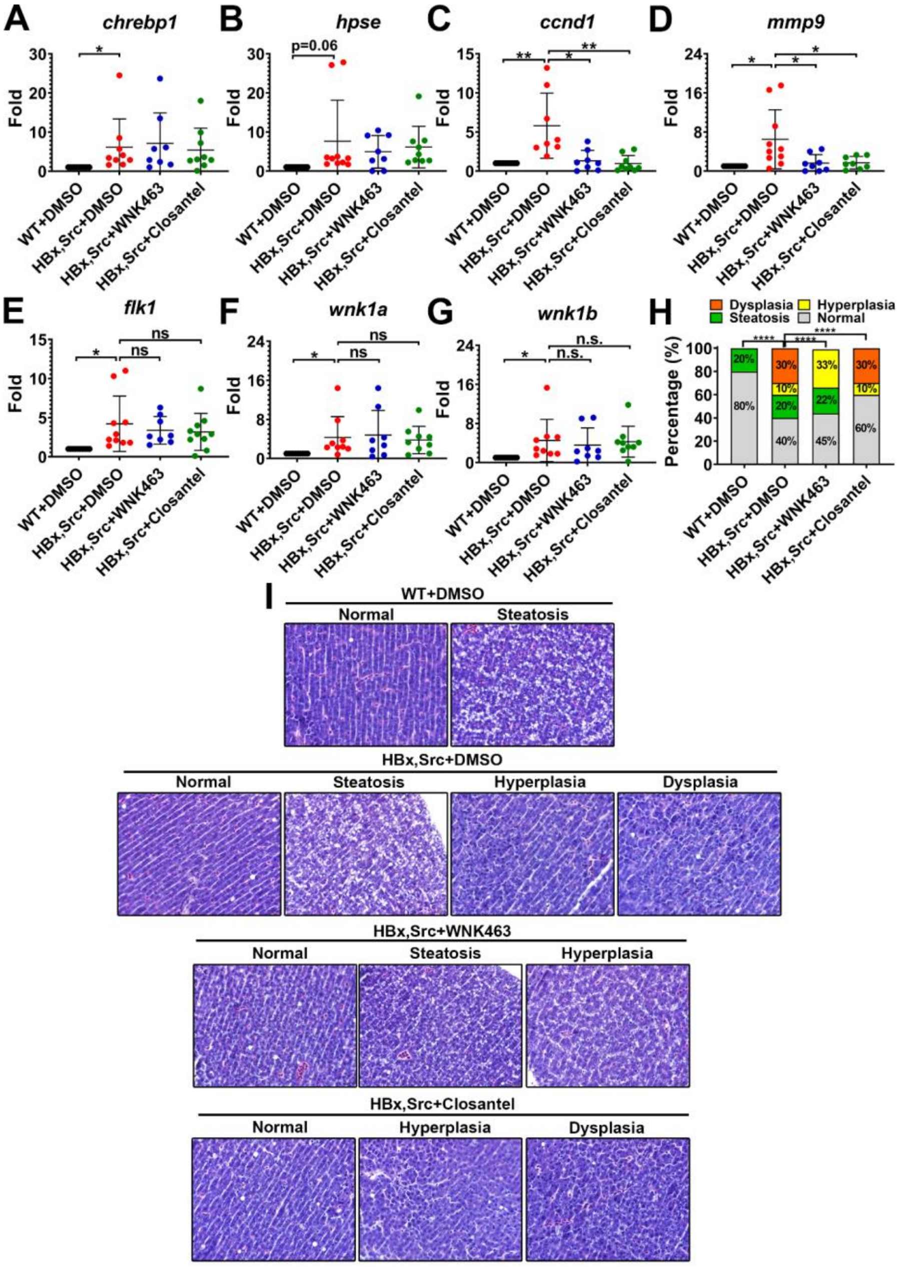

Figure S5. Effect of WNK1 pathway inhibitors on hepatocellular carcinoma (HCC) in transgenic fish. (**A-G**) QPCR analysis of lipogenic factor carbohydrate-responsive element-binding protein (*chrebp1*), fibrosis maker gene heparanase (*hpse*), cell cycle gene cyclin D1 (*ccnd1*), matrix metallopeptidase 9 (*mmp9*), VEGF receptor-2 (*flk1/vegfr2*), zebrafish WNK1 orthologue (*wnk1a* and *wnk1b*) in HCC transgenic fish treated with DMSO (n = 10), WNK463 (n =9) or Closantel (n = 10) were

Formatted: Font: Not Bold

Formatted: Font: Not Bold

performed and compared to wildtype fish treated with DMSO (n = 10). *: 0.01<P≤0.05; **: 0.001<P≤0.01; ***: P≤0.001. (**H**) Distribution of histopathological findings and (**I**) representative images and of H&E-stained liver sections from WT and transgenic fish treated with DMSO, WNK463 or Closantel. Scale bar = 20 μm.

Reference

1. Muller, P.Y.; Milton, M.N. The determination and interpretation of the therapeutic index in drug development. *Nat Rev Drug Discov* **2012**, *11*, 751-761, doi:10.1038/nrd3801.
